# A Rare Presentation of Zoledronate-Induced Systemic Inflammatory Response

**DOI:** 10.7759/cureus.41524

**Published:** 2023-07-07

**Authors:** Maria Jamil, Amir Daneshvar, Dana Nachawati, Husam El Sharu, Alireza Meysami

**Affiliations:** 1 Internal Medicine, Henry Ford Health System, Detroit, USA; 2 Internal Medicine, East Carolina University, Greenville, USA; 3 Rheumatology, Henry Ford Health System, Detroit, USA

**Keywords:** bisphosphonates, elderly patients, immune mediated reaction, osteoporosis management, zoledronic acid, internal medicine and rheumatology

## Abstract

Zoledronic acid is a bisphosphonate commonly used to treat various conditions involving bone loss. While it is generally well-tolerated, the occurrence of severe inflammatory reactions is rare. We present the case of an 82-year-old female who developed a severe immune reaction, including weakness and tenderness in her upper and lower extremities, following a single dose of zoledronic acid infusion for the treatment of osteoporosis. The onset of symptoms occurred one week after the infusion and persisted, progressively worsening over time, leading to functional impairment and the need for a walker for ambulation. Laboratory studies revealed an elevated erythrocyte sedimentation rate while other autoimmune markers were within normal limits. Differential diagnosis included an adverse reaction to zoledronic acid or underlying polymyalgia rheumatica. The patient showed significant improvement with a prednisone taper, suggesting an immune-mediated response. This case highlights the importance of considering severe immune reactions as a potential side effect of zoledronic acid and emphasizes the need for further research to better understand the underlying mechanisms and optimize patient management.

## Introduction

Zoledronic acid belongs to a class of medications known as bisphosphonates, which work by slowing down bone breakdown, increasing bone density, and reducing the release of calcium from bones into the bloodstream [1]. Common side effects of zoledronic acid include fever, chills, mild joint pain, headaches, and myalgias. These side effects typically last for 24-72 hours and do not require treatment. Although mild arthralgias are a common side effect, there have been only two previously reported studies of zoledronic acid causing a severe inflammatory response [2]. In this case report, we present the case of an 82-year-old female who experienced a severe immune reaction, including pronounced weakness and tenderness in her upper and lower extremities, one week after zoledronic acid administration. 

## Case presentation

An 82-year-old female presented to the rheumatology clinic with a primary complaint of progressive weakness and tenderness in her bilateral upper extremities, including her hands, arms, and shoulders. Additionally, she experienced worsening gait disturbances, which led her to rely on a walker for ambulation. The patient had a history of osteoporosis diagnosed through a prior dual X-ray absorptiometry (DEXA) scan. In September 2022, she was referred for zoledronic acid infusion as a treatment for her osteoporosis. Prior to the infusion, she had complete mobility independence and did not require any assistive devices for walking. 

Around one week after the infusion, the patient began noticing joint pain and weakness in both her upper and lower extremities. A month later, she visited her primary care physician who advised her to maintain adequate hydration, assuring her that the symptoms should improve within a couple of weeks as myalgias and arthralgias are common side effects of the infusion. However, three months after the infusion, she returned to her primary care physician, reporting progressively worsening symptoms and unbearable pain. At that time, laboratory studies were conducted, including an erythrocyte sedimentation rate (ESR), aldolase, C-reactive protein (CRP), and creatine phosphokinase (CPK). The ESR was elevated to 62 mm/hr, while CRP, aldolase, and CPK were within normal limits. Subsequently, she was referred to rheumatology for further evaluation. 

Seven months after the infusion, the patient presented to the rheumatology clinic and stated that her symptoms had continued to worsen and she now relied on a walker for ambulation. She denied the development of any malar rashes, oral sores, Raynaud's features, or photosensitivity. Despite trying various pain medications such as diclofenac gel, cold packs, and cannabidiol creams, she experienced no significant improvement in her symptoms. Examination was positive for joint erythema and tenderness affecting mainly her distal and shoulder joints as well her interphalangeal and metacarpophalangeal joints of her hands and feet. These joints were painful on both active and passive movement. The differential diagnosis included a possible underlying rheumatoid arthritis, considering her family history of rheumatoid arthritis in her sister, as well as a positive antinuclear antibody (ANA) test in 2017. Polymyalgia rheumatica (PMR) was also considered a possibility. 

An autoimmune workup was conducted (shown in Table 1) and her anti-double stranded DNA (anti-dsDNA) antibody, C4 and C3 complement, ribonucleoprotein antibody, anti-Smith antibody, rheumatoid factor, Sjögren's-syndrome-related antigen A&B and her anti-cyclic citrullinated peptide were all negative. Currently, the patient is undergoing a five-week prednisone taper, which has resulted in significant improvement in her pain and range of motion in the upper extremities. 

**Table 1:** Autoimmune panel Anti-dsDNA: Anti-double-stranded deoxyribonucleic acid C3/C4: Complement 3/complement 4 RNP: Ribonucleoprotein SM: Smith RF: Rheumatoid factor SS A/B: Sjögren's-syndrome-related antigen A&B Anti-CCP: Anti-cyclic citrullinated peptide

Anti-dsDNA antibody	C4 complement	C3 complement	RNP antibody	SM antibody	RF	SS A/B/Ro antibody	Anti-CCP antibody
Negative	32 mg/dL (10 - 51 mg/dL)	134 mg/dL (90 - 230 mg/dL)	<0.2 Elisa Units (<1.0 Elisa Units)	<0.2 Elisa Units (<1.0 Elisa Units)	<10 IU/mL (<14 IU/mL)	Negative	0.7 IU/mL (<7 IU/mL)

## Discussion

Bisphosphonates, including zoledronic acid, are widely used as the primary agents to counteract osteoclast-mediated bone loss in various conditions such as multiple myeloma, hypercalcemia of malignancy, Paget's disease, and osteoporosis. These medications are chemical derivatives of inorganic pyrophosphate [3]. However, due to common side effects such as gastrointestinal and esophageal irritation, as well as the complex administration methods, oral bisphosphonates have been associated with low patient compliance rates compared to zoledronic acid infusions. Studies have reported a compliance rate of only around 35-45% per year [4]. 

An important step in the process of new bone formation involves the binding of inorganic pyrophosphate (PPi) to hydroxyapatite crystals [5]. Zoledronate, a nitrogen-containing bisphosphonate, is an analog of PPi with a higher binding affinity. It can selectively bind to actively remodeling bone sites. The bisphosphonates bound to bone are released during the process of bone breakdown by osteoclasts [6]. Zoledronic acid, being a chemical derivative, can be administered as a yearly 5 mg intravenous infusion. Studies have shown that it can increase bone mineral density and decrease bone turnover over time, ultimately reducing the risk of fractures [6]. 

Typical reported side effects of zoledronic acid include mild arthralgias, myalgias, and fever. However, these side effects have been reported as self-limiting and manageable in the outpatient setting, lasting approximately 48-72 hours [7]. In our patient, however, her symptoms persisted beyond this timeframe and were more severe than what previous studies have reported, accompanied by a significant elevation in her ESR and functional impairment. We identified only two other case reports with similar findings [8-9]. 

While the patient's symptoms, including weakness and tenderness in the upper extremities, initially resembled those of PMR, several factors argue against a diagnosis of PMR in this case. Firstly, the temporal relationship between symptom onset and zoledronic acid infusion raises the possibility of an adverse drug reaction rather than an underlying PMR. Furthermore, the patient's laboratory findings, particularly the elevated ESR, could be attributed to the immune reaction triggered by zoledronic acid rather than indicative of PMR. Considering these factors, an adverse reaction to zoledronic acid appears to be the more plausible explanation for the patient's symptoms rather than an underlying PMR.

Following the administration of bisphosphonates, it has been reported that higher levels of interleukin-6 (IL-6) and tumor necrosis factor (TNF) contribute to the development of transient fevers and pyrexia, usually lasting up to three days [8]. The release and proliferation of gamma-delta T lymphocytes are believed to be involved in this process, although the exact mechanisms are not well understood [8]. We hypothesize that the release of proinflammatory cytokines is the most probable cause of her immune reaction. 

Gamma-delta T lymphocytes represent a subset of T cells that express a unique T cell receptor (TCR) composed of gamma and delta chains. These cells are part of the innate immune response and are found in various tissues, including the skin, gut, and lungs. It has been proposed that the activation of gamma-delta T lymphocytes plays a role in the development of arthritic symptoms following zoledronic acid infusion, leading to the release of cytokines that induce inflammation and joint pain [8,10]. The activation of gamma-delta T lymphocytes is considered a crucial factor in the acute phase reaction (APR) observed after zoledronic acid infusion [10]. Musculoskeletal side effects, including musculoskeletal pain, new arthritis, painful joints, and joint swelling, have also been reported following intravenous administration of zoledronic acid [11]. 

## Conclusions

This case emphasizes the significance of recognizing severe immune reactions as potential adverse effects of zoledronic acid and stresses the necessity for additional research to enhance our understanding of the underlying mechanisms and improve patient care. It highlights the importance of healthcare providers being aware of the potential for severe side effects related to zoledronic acid infusion. Further investigation is required to explore potential connections between immune responses and bisphosphonates. 
